# Complete genomes of two *Chlamydia psittaci* isolated from ducks and pigeons in China

**DOI:** 10.1128/mra.00606-25

**Published:** 2025-10-10

**Authors:** Xiaoxue Wang, Yihan Wang, Yanyan Wang, Junkai Zhang, Chunguo Liu, Ping Liu, Zhaocai Li, Jizhang Zhou

**Affiliations:** 1 State Key Laboratory of Animal Diseases Control and Prevention, Lanzhou Veterinary Research Institute, Chinese Academy of Agricultural Sciences 111658, Lanzhou, China; 2 College of Advanced Agricultural Sciences, Yulin University, Yulin, Shaanxi, China; 3 Group Testing Center, Shandong Sinder Technology Co. Ltd, Qingdao, China; Montana State University, Bozeman, Montana, USA

**Keywords:** *Chlamydia psittaci*, genome, psittacosis

## Abstract

*Chlamydia psittaci* is an important zoonotic pathogen that can transmit from avian to human. Herein, we report complete genome sequences of two *C. psittaci* strains isolated from ducks and pigeons in China. The duck strain CPS-QD/LS was identified as *ompA* genotype A, while the pigeon strain CPS-BY/JY was identified as genotype B.

## ANNOUNCEMENT

*Chlamydia psittaci* is a unique zoonotic intracellular bacterial pathogen that can infect avian species, mammals, and humans (1, 2). Based on *ompA* gene sequences, the *C. psittaci* isolates can be classified into nine classical (A to F, E/B, M56, and WC) and several atypical genotypes (3). Previous studies have shown that *C. psittaci* infections in avian species are common in China. However, few genome data are available for analysis of its genetic diversity and host specificity. During routine detection of *Chlamydia* species in our laboratory, genomic DNA was isolated from pure cultures of *C. psittaci* using the TIANamp Genomic DNA Kit (TIANGEN, Beijing) (4). We isolated two *C. psittaci* strains from real-time PCR-positive duck lung tissue and pigeon cloacal swab samples by inoculating the samples into specific-pathogen-free embryonated chicken eggs (5). The isolates obtained were confirmed by real-time PCR (6) and were named as CPS-QD/LS (duck strain) and CPS-BY/JY (pigeon strain) according to their host origin region. The isolates were then inoculated into the L929 cell line for propagation and purification for genome sequencing.

Purified genome DNA was prepared for sequencing using a combination of Illumina NovaSeq 6000 and Nanopore PromethION platforms. The Illumina library was prepared using the TruSeq DNA Library Prep Kit (Illumina, San Diego, USA) and amplified within the flow cell on an Illumina cBOT instrument for cluster generation (NovaSeq 6000 PE Cluster Kit, Illumina). The clustered flow cell was loaded onto a NovaSeq 6000 sequencer (Illumina) for paired-end sequencing. The purified libraries were loaded onto primed R9.4 Spot-On flow cells and sequenced on a PacBio sequencer (Pacific Biosciences, Menlo Park, USA). Raw reads were filtered with fastp (v.0.23.2) (7), yielding 929,758,353 and 1,829,539,148 bp of clean data for strains CPS-QD/LS and CPS-BY/JY, respectively. In parallel, libraries were prepared using the NEBNext Ultra II DNA Library Prep Kit (NEB, USA) and sequenced on the PromethION platform (Oxford Nanopore Technologies, Oxford, UK) (8). Raw reads were quality-filtered (*Q* ≥ 7) and length-filtered (≥1,600 bp), generating 1,087,063,417 and 2,137,810,282 bp of pass reads for CPS-QD/LS and CPS-BY/JY, respectively, with N50 values of 9.04 and 5.55 kb. Hybrid genome assembly of Illumina and Nanopore reads was performed using SOAPdenovo (v.2) (9). The repeat sequence analysis was performed using the software RepeatMasker (4.1.2-p1) (10). Circularization of contigs and re-orientation to the dnaA start were assessed and corrected using BLAST. Gene prediction was carried out with Prokka (v.1.14.6) (11). Promoter regions were predicted using PromPredict (v.V1) (12), and transmembrane proteins were identified with TMHMM (v.2.0c) (13). All software was run with default parameters.

**Fig 1:**
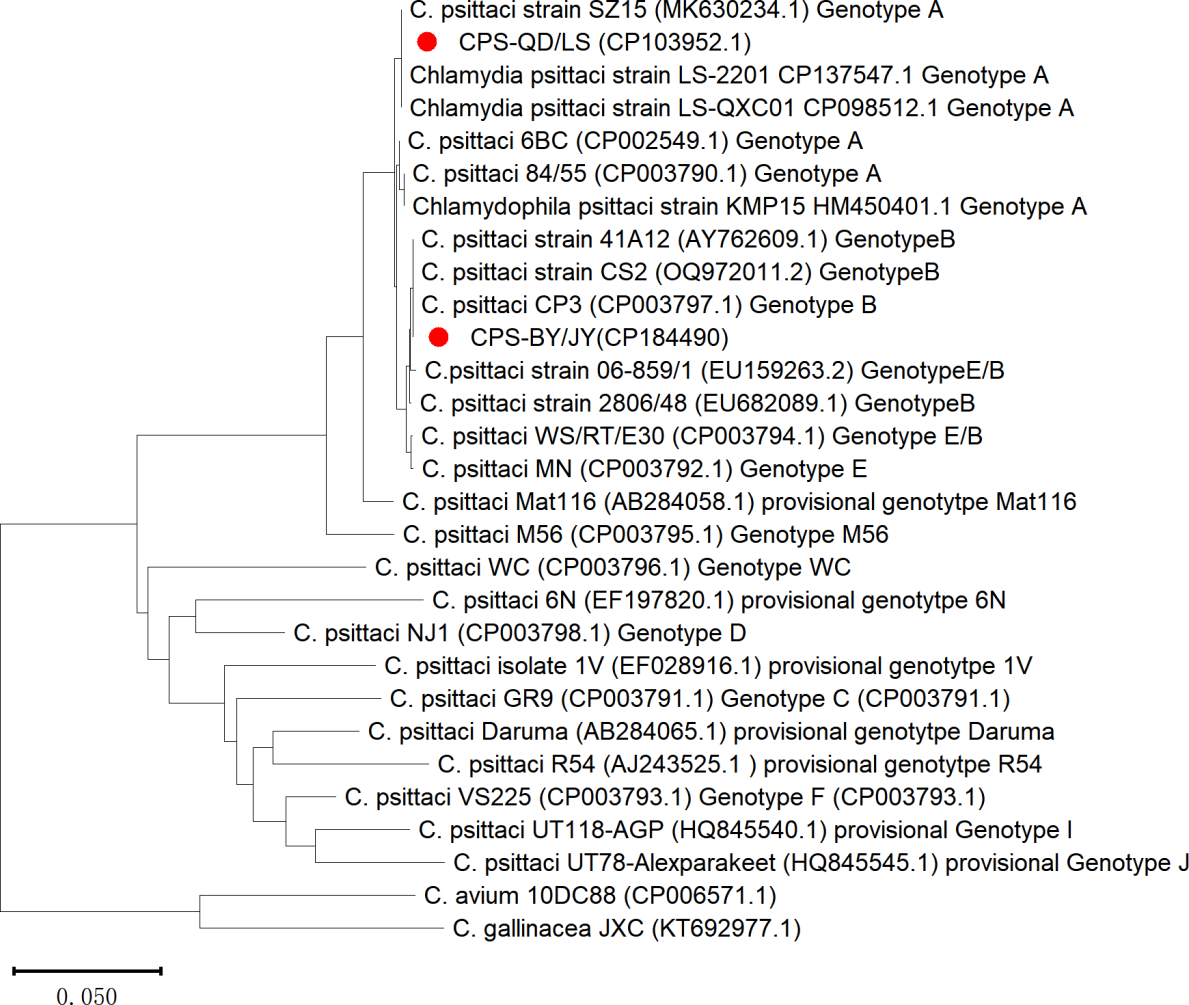
Clustering analysis of *Chlamydia psittaci ompA* genes.

The final genomes of *C. psittaci* were complete, and both genomes comprised a circular chromosome and a plasmid, with GenBank accession numbers CP103952.1, CP103953.1, CP184490.1, and CP184489.1. The genomes were annotated by National Center for Biotechnology Information Prokaryotic Genome Annotation Pipeline (v.6.2/v.6.9). The genome characteristics are summarized in Table 1. Sequence analysis of the *ompA* gene of the two obtained isolates showed that duck strain CPS-QD/LS was identical to that of a duck isolate, SZ15, and two human isolates in China (14), which could be classified to the *ompA* genotype A group (Fig. 1), while the *ompA* gene sequence of CPS-BY/JY matched the *C. psittaci* strains of the typical genotype B group (15), as shown in Fig. 1. These data may improve our understanding of the genetic diversity and host specificity of this *Chlamydia* species.

**TABLE 1:** Sample isolation data and genome characteristics

	CPS-QD/LS		CPS-BY/JY	
Parameters	Chromosome	Plasmid	Chromosome	Plasmid
GenBank accession no.	CP103952.1	CP103953.1	CP184490.1	CP184489.1
Clean data (bp)	1,087,063,417 (Nanopore)		2,137,810,282 (Nanopore)	
	929,758,353 (Illumina)		1,829,539,148 (Illumina)	
Read length for Nanopore (bp)	174,286 (N50 9.04 kb)		473,993 (N50 5.55 kb)	
Read length for PacBio (bp)	6,339,706		12,381,778	
Contig length (bp)	1,149,432	7,553	1,168,334	7,553
G + C content (%)	39.08	32.95	39.05	32.82
Sequencing depth (×)	392.5 (Nanopore)		486.89 (Nanopore)	
	468.04 (Illumina)		305.58 (Illumina)	
No. of pseudogenes	79	0	88	0
No. of tRNAs	39	0	39	0
No. of rRNAs	3	0	3	0
No. of tmRNAs	3	0	1	0
No. of misc_RNA	3	0	2	0
SRA accession no.	SRR33065692		SRR33068449	

## Data Availability

The chromosomal and plasmid genome sequences of Chlamydia psittaci isolates CPS-QD/LS and CPS-BY/JY have been deposited in GenBank under accession nos. CP103952.1, CP103953.1, CP184490.1, and CP184489.1. The original sequence reads are deposited in the Sequence Read Archive under accession numbers SRR33065692 and SRR33068449.

## References

[1] Kaleta EF, Taday EMA. 2003. Avian host range of Chlamydophila spp. based on isolation, antigen detection and serology. Avian Pathol 32:435–461. doi:10.1080/03079450310001593613 14522700

[2] Li Z, Liu P, Hou J, Xu G, Zhang J, Lei Y, Lou Z, Liang L, Wen Y, Zhou J. 2020. Detection of Chlamydia psittaci and Chlamydia ibidis in the endangered crested ibis (Nipponia nippon). Epidemiol Infect 148:1–5. doi:10.1017/S0950268819002231 PMC7019082 31910921

[3] Ravichandran K, Anbazhagan S, Karthik K, Angappan M, Dhayananth B. 2021. A comprehensive review on avian chlamydiosis: a neglected zoonotic disease. Trop Anim Health Prod 53:414. doi:10.1007/s11250-021-02859-0 34312716 PMC8313243

[4] TIANamp Genomic DNA Kit. n.d. Available from: https://en.tiangen.com/content/details_156_8223.html

[5] Pantchev A, Sting R, Bauerfeind R, Tyczka J, Sachse K. 2010. Detection of all Chlamydophila and Chlamydia spp. of veterinary interest using species-specific real-time PCR assays. Comp Immunol Microbiol Infect Dis 33:473–484. doi:10.1016/j.cimid.2009.08.002 19733907

[6] Braukmann M, Sachse K, Jacobsen ID, Westermann M, Menge C, Saluz HP, Berndt A. 2012. Distinct intensity of host-pathogen interactions in Chlamydia psittaci- and Chlamydia abortus-infected chicken embryos. Infect Immun 80:2976–2988. doi:10.1128/IAI.00437-12 22689815 PMC3418749

[7] Chen S, Zhou Y, Chen Y, Gu J. 2018. Fastp: an ultra-fast all-in-one FASTQ preprocessor. Bioinformatics 34:i884–i890. doi:10.1093/bioinformatics/bty560 30423086 PMC6129281

[8] Kolmogorov M, Billingsley KJ, Mastoras M, et al.. 2023. Scalable Nanopore sequencing of human genomes provides a comprehensive view of haplotype-resolved variation and methylation. Nat Methods 20:1483–1492. doi:10.1038/s41592-023-01993-x 37710018 PMC11222905

[9] Li R, Zhu H, Ruan J, Qian W, Fang X, Shi Z, Li Y, Li S, Shan G, Kristiansen K, Li S, Yang H, Wang J, Wang J. 2010. De novo assembly of human genomes with massively parallel short read sequencing. Genome Res 20:265–272. doi:10.1101/gr.097261.109 20019144 PMC2813482

[10] Tarailo-Graovac M, Chen N. 2009. Using RepeatMasker to identify repetitive elements in genomic sequences. Curr Protoc Bioinform Chapter 4:4. doi:10.1002/0471250953.bi0410s25 19274634

[11] Seemann T. 2014. Prokka: rapid prokaryotic genome annotation. Bioinformatics 30:2068–2069. doi:10.1093/bioinformatics/btu153 24642063

[12] Huerta CJ, Serra BM, Gabaldón T. 2019. eggNOG 5.0: a hierarchical, functionally - and phylogenetically - aware orthology resource for bacteria and archaea. Nucleic Acids Res 47:D350–D359. doi:10.1093/nar/gky1085 PMC6324079 30418610

[13] Krogh A, Larsson B, von Heijne G, Sonnhammer EL. 2001. Predicting transmembrane protein topology with a hidden Markov model: application to complete genomes. J Mol Biol 305:567–580. doi:10.1006/jmbi.2000.4315 11152613

[14] Lin W, Chen T, Liao L, Wang Z, Xiao J, Lu J, Song C, Qin J, Chen F, Chang YF, Xie Q. 2019. A parrot-type Chlamydia psittaci strain is in association with egg production drop in laying ducks. Transbound Emerg Dis 66:2002–2010. doi:10.1111/tbed.13248 31127977

[15] Mitchell SL, Wolff BJ, Thacker WL, Ciembor PG, Gregory CR, Everett KDE, Ritchie BW, Winchell JM. 2009. Genotyping of Chlamydophila psittaci by real-time PCR and high-resolution melt analysis. J Clin Microbiol 47:175–181. doi:10.1128/JCM.01851-08 19005152 PMC2620869

